# Supplementary material for: Case Report: Identification of a Novel Variant (m.8909T>C) of Human Mitochondrial ATP6 Gene and Its Functional Consequences on Yeast ATP Synthase
Source: Life (Basel). 2020 Sep 22;10(9):215. doi: 10.3390/life10090215 (PMC7555451; doi:10.3390/life10090215)
Supplement: Supplementary file 1 [file life-10-00215-s001.pdf]

life

MDPI

Supplemental Information

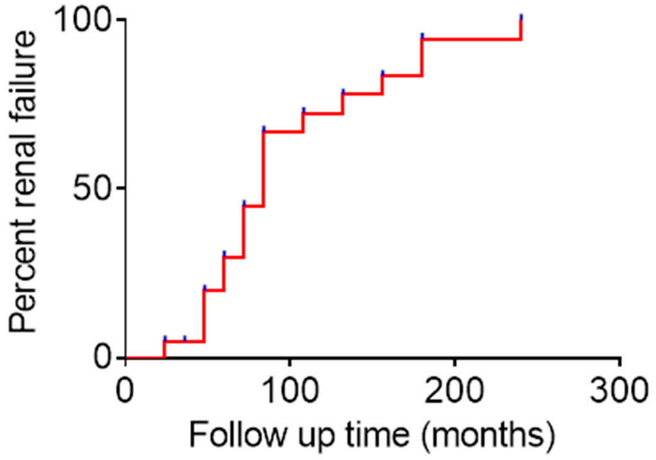

Figure S1. Occurrence of renal failure during follow-up of 35 previously reported m.3243G>A patients diagnosed with a nephrotic syndrome.

Table S1. Mean clinical characteristics of 35 previously reported m.3243G>A, patients diagnosed with a nephrotic syndrome.

**Mitochondrial Disease with Kidney Involvement**	
Reported patients	N = 35
Onset age of renal disease (years)	26 (6.0–59.0)
Male (%)	17 (48.6%)
Family history	29 (82.9%)
Proteinuria	19 (54.3%)
Pathology feature	N = 21
FSGS	15 (71.4%)
TIN	3 (14.3%)
Follow-up (months)	N = 21<br>72.0 (24.0–240.0)
Poor Outcome[a]	19 (90.5%)
Onset age of end stage of kidney disease or death (years)	37 (13.0–51.0)

[a] Poor outcome means end stage of kidney disease or death at the end of follow-up. Abbreviations: TIN, tubulointerstitial nephropathy; FSGS, Focal segmental glomerulosclerosis (FSGS).

Table S2. Individual clinical characteristics of 35 previously reported m.3243G>A patients diagnosed with a nephrotic syndrome.

Source	Gender	Onset age of renal disease (yrs)	Renal features	Renal histology	Other clinical features	Family history	Genotype
[59]	F	45	proteinuria	FSGS	MELAS, hearing loss, DM	Positive	A3243G
[59]	M	38	renal insufficiency, hemodialysis	FSGS	MELAS, hearing loss, DM	Positive	A3243G
[60]	F	18	proteinuria, edema, renal dysfunction	FSGS	MELAS	Negative	A3243G
[61]	M	18	NS, hematuria, renal failure at age of 24	FSGS	DM, neurosensory deafness and cataract	Positive	A3243G
[62]	F	31	NS, and then progressive renal failure	FSGS	DM, hearing loss, concentric left ventricular hypertrophy	Positive	A3243G
[63]	F	47	NS, ESRD at age 51	FSGS	hypertrophic, DM, cardiomyopathy and sensorineural deafness	Negative	A3243G
[64]	M	23	proteinuria, renal failure at the age of 38	FSGS	sensorineural hearing loss, DM	Positive	A3243G
[65]	M	25	proteinuria	glomerular collapses and thickening of the glomerular basement membrane	MELAS	Negative	A3243G
[66]	M	37	proteinuria, hemodialysis at 46 years old		MELAS	Negative	A3243G
[63]	F	41	proteinuria, and renal failure at 47 years old	TIN	neurosensory deafness, cardiomyopathy, DM, transient ischemic stroke, macular dystrophy	Positive	A3243G
[63]	M	27	proteinuria, renal failure at the age of 33	TIN	neurosensory deafness, DM, hypertrophic cardiomyopathy	Positive	A3243G
[67]	M	32	progressive renal failure		MELAS, DM, left ventricular hypertrophy	Positive	A3243G
[68]	F		progressive renal failure with proteinuria		MELAS, cardiomyopathy, DM, hyperthyroidism	Positive	A3243G
[69]	F	26	proteinuria, and then progressive renal failure at age of 33	chronic glomerulosclerosis	DM, hearing loss	Positive	A3243G
[69]	F	26	proteinuria, and then progressive renal failure at age of 33	chronic lobular glomerulonephritis	DM, hearing loss	Positive	A3243G
[69]	F	26	proteinuria, and then progressive renal failure at age of 33	FSGS	DM, hearing loss	Positive	A3243G
[70]	M	48	renal insufficiency		MELAS, deafness	Negative	A3243G
[59]	M	19	renal failure, dialysis at 26 years of age		MELAS	Positive	A3243G
[59]	M	44	renal failure, ESRD at 48 years old		MELAS	Negative	A3243G
[63]	M	32	proteinuria, ESRD at age 36 years old		muscle fatigability, sensorineural deafness,DM, cardiomyopathy	Positive	A3243G
[71]	M	21	proteinuria, ESRD at 41 years old		hearing loss, DM, Wolff-Parkinson-White arrhythmia	Positive	A3243G
[72]	M	59	renal failure at age of 64		cardiomyopathy, sensorineural deafness, DM	Positive	A3243G
[73]	M		renal failure		cardiomyopathy, DM, deafness, headache	Positive	A3243G
[74]	F	49	renal failure		cardiomyopathy, hepatic dysfunction, deafness	Positive	A3243G
[75]	F	46	ESRD and hemodialysis		DM, sensorineural hearing loss	Positive	A3243G
[63]	F	41	proteinuria and ESRD		sensorineural deafness, DM	Positive	A3243G

[76]	M	25	chronic renal failure at age of 40		MELAS	Positive	A3243G
[77]	M	6	proteinuria	TIN	Cataract	Positive	A3243G
[78]	F	12	NS, facial edema, renal failure and peritoneal dialysis at age of 14	FSGS	MELAS, Alport syndrome, DN	Positive	A3243G
[79]	M	13	proteinuria	FSGS	DM, growth hormone deficiency	Positive	A3243G
[80]	F	10	proteinuria	FSGS	short in stature	Positive	A3243G
[80]	F	17	proteinuria	FSGS	short in stature	Positive	A3243G
[80]	F	14	proteinuria, Renal failure at age of 25	FSGS	short in stature	Positive	A3243G
[80]	F	15	proteinuria, Dialysis at age of 28	FSGS	hearing disturbance, short in stature	Positive	A3243G
[81]	F	14	proteinuria	FSGS	DM, hearing loss and mild hypothyroidism, growth retardation	positive	A3243G
**The Patient in Our Study**							
	F	14	NS, ESRD and died at age 14	MPGN	MELAS, hemiplegia, epileptic episodes, aphasia, blindness and deafness, hyper lactacidemia	Negative	A3243GT8909C

ESRD, Kidney failure or end-stage renal disease; MELAS, mitochondrial encephalomyopathy lactic acidosis and stroke-like episodes; TIN, tubulointerstitial nephropathy; FSGS, Focal segmental glomerulosclerosis.
